# Interplay of thermal diffuse scattering and correlated compositional disorder in KCl_1–*x*_Br_*x*_

**DOI:** 10.1107/S2052520622003560

**Published:** 2022-05-11

**Authors:** Ella Mara Schmidt, Sofia Thomas, Johnathan M. Bulled, Arianna Minelli, Andrew L. Goodwin

**Affiliations:** a Inorganic Chemistry Laboratory, University of Oxford, South Parks Road, OX1 3QR, United Kingdom; b Faculty of Geosciences, MARUM and MAPEX, University of Bremen, Klagenfurter Str. 2-4, 28359 Bremen, Germany

**Keywords:** disordered rocksalts, diffuse scattering, lattice dynamics

## Abstract

Single-crystal X-ray diffuse scattering measurements are reported of the compositional series KCl_1–*x*_Br_*x*_, a model system for the broader family of disordered rocksalts. Using a combination of Monte Carlo simulations and lattice dynamical calculations, we show that the observed diffuse scattering is well described in terms of (i) non-statistical anion distributions, (ii) local lattice relaxations accompanying Cl/Br substitution, and (iii) the contribution from low-energy phonons. It is found that a tendency for compositional domain formation broadens the thermal diffuse scattering by splitting and softening the acoustic phonon branches. This effect, which is strongest for intermediate compositions, is seen in both experiment and calculation alike. These results establish a link between local compositional order and unconventional lattice dynamics in this system, and reinforce emerging design principles of exploiting compositional fluctuations to tailor physical properties, such as thermal conductivity, that depend on phonon broadening.

## Abstract 2

Single-crystal diffuse scattering measurements are used to study the interplay of short-range anion order and anharmonic lattice dynamics in the series KCl_1–*x*_Br_*x*_.

## 1. Introduction

The interplay of compositional disorder and lattice dynamics is a longstanding and notoriously difficult problem (Klemens, 1960 ▸; Callaway & von Baeyer, 1960 ▸; Ziman, 1979 ▸; Seyf *et al.*, 2017 ▸) of particular currency in the fields of photoluminescence, photovoltaics and thermoelectric materials design. For example, in the series [CH_3_NH_3_]Pb(Br_*x*_I_1–*x*_)_3_ (Tombe *et al.*, 2017 ▸), photoluminescence lifetimes are controlled by exciton–phonon coupling, which in turn is affected by anion distributions (Feldmann *et al.*, 2020 ▸). Likewise, a tendency for spinodal decomposition in the PbTe–PbS system drives complex nanostructures in PbTe_1–*x*_S_*x*_ phases (Kageyama *et al.*, 2018 ▸; Snyder & Toberer, 2008 ▸; Zeuthen *et al.*, 2022 ▸) that reduce thermal conductivity; this approach has been exploited to achieve a large thermoelectric figure of merit (*ZT* > 2) in PbTe_0.7_S_0.3_ (Wu *et al.*, 2014 ▸).

From both experimental and theoretical viewpoints, the particular challenge faced is that the correlations present in compositionally disordered solids can influence the way in which such disorder couples to the lattice dynamics (Ziman, 1979 ▸; Overy *et al.*, 2016 ▸). At one extreme, the so-called ‘virtual crystal’ limit, the form of the phonon dispersion is insensitive to compositional variation, with the dispersion relation well described by interpolation between those of the endmember compositions (Abeles, 1963 ▸; Seyf *et al.*, 2017 ▸). At the other extreme, dopants behave as independent oscillators, as in *e.g.* Fe_1–*x*_Ir_*x*_Si (Delaire *et al.*, 2015 ▸), resulting in the superposition of a dispersionless ‘resonance’ on the phonon spectrum of the parent compound. The trickiest cases are those in between, for which the lattice dynamics are described neither by interpolation nor by superposition from the two compositionally-ordered endmembers (Mattis & Bardeen, 1958 ▸; Kamitakahara & Brockhouse, 1974 ▸).

Here we explore the sensitivity of single-crystal X-ray diffuse scattering measurements to the interplay of compositional disorder and lattice dynamics in rocksalt-structured KCl_1–*x*_Br_*x*_. Our choice of system was motivated by the importance of compositional disorder on the f.c.c. lattice to a wide range of systems, from disordered rocksalt cathodes (Chen *et al.*, 2021 ▸; Clément *et al.*, 2020 ▸) to relaxor ferroelectrics (Paściak *et al.*, 2012 ▸). In this context, KCl_1–*x*_Br_*x*_ is prototypical as it intentionally avoids the additional complexity associated with *e.g.* charge order or Jahn–Teller instabilities found in many functional rocksalt oxides. Likewise, our choice of experiment was informed by the fact that energy-integrated diffuse scattering is known to contain contributions from compositional disorder, correlated displacements (*i.e.* phonons) and the coupling between compositional variation and atomic displacements (Welberry & Weber, 2016 ▸; Welberry & Butler, 1994 ▸). Of course, the use of crystallographic experiments to probe dynamical effects has a long and important history (Bürgi & Dunitz, 1983 ▸; Bürgi & Capelli, 2000 ▸; Bürgi, 2000 ▸).

Anticipating our results, we will come to show that the experimental X-ray diffuse scattering patterns of KCl_1–*x*_Br_*x*_ imply anion clustering, *i.e.* the formation of Cl^−^- and Br^−^-rich domains, and that this clustering broadens the acoustic phonons that dominate the thermal diffuse scattering. In this way, the thermal diffuse scattering, although inelastic in origin, is itself sensitive to the (static) compositional ordering of Cl^−^ and Br^−^ ions.

Our paper is organised as follows. We begin by presenting our experimental diffuse scattering measurements, then proceed to describe a series of Monte Carlo KCl_1–*x*_Br_*x*_ simulations that span both composition *x* and tendency for anion (anti)clustering. From these configurations, and using established lattice dynamical models for KCl/KBr, we calculate the corresponding single-crystal X-ray diffuse scattering patterns and establish the best match to experiment. We conclude by discussing the implications of local compositional order for the lattice dynamics of KCl_1–*x*_Br_*x*_ specifically, and ultimately with reference to the more general problem of exploiting disorder to control the lattice dynamics of various key classes of functional materials.

## 2. Results and discussion

### 2.1. Synthesis and characterisation

We prepared KCl_1–*x*_Br_*x*_ samples in both single-crystal and polycrystalline forms using a single common approach. Aqueous solutions of KBr and KCl (1 M; Sigma Aldrich) were combined in appropriate proportions for a given stoichiometry *x*. The resulting solution was then allowed to evaporate to dryness, and the crystals so produced harvested. Polycrystalline samples for the eleven compositions *x* = 0, 0.1, …, 0.9, 1 were ground into a fine powder and their X-ray diffraction patterns measured. The corresponding unit-cell parameters, determined using Pawley refinements, obeyed Vegard’s law and supported the assumption that nominal and actual compositions coincide [see supporting information for further discussion]. Single crystal samples suitable for diffuse scattering measurements were obtained for the five compositions *x* = 0, 0.2, 0.5, 0.8, 1.

### 2.2. Diffuse scattering measurements

Single-crystal X-ray diffuse scattering measurements were performed on a Rigaku Synergy S diffractometer equipped with an Eiger 1M detector and using Cu radiation. The data acquisition strategy was chosen to maximise reciprocal space coverage, detailed information is given in the supporting information. The software *CrysAlisPRO* (Agilent, 2014 ▸) was used to determine the unit-cell constants and orientation matrices. In all cases, the expected 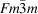 reflection conditions were obeyed. We did not observe any obvious differences in mosaicity for the various samples. The three-dimensional scattering function (Bragg + diffuse) for each crystal was reconstructed and symmetry-averaged using the *Meerkat* software (Simonov, 2020 ▸).

**Figure 1:**
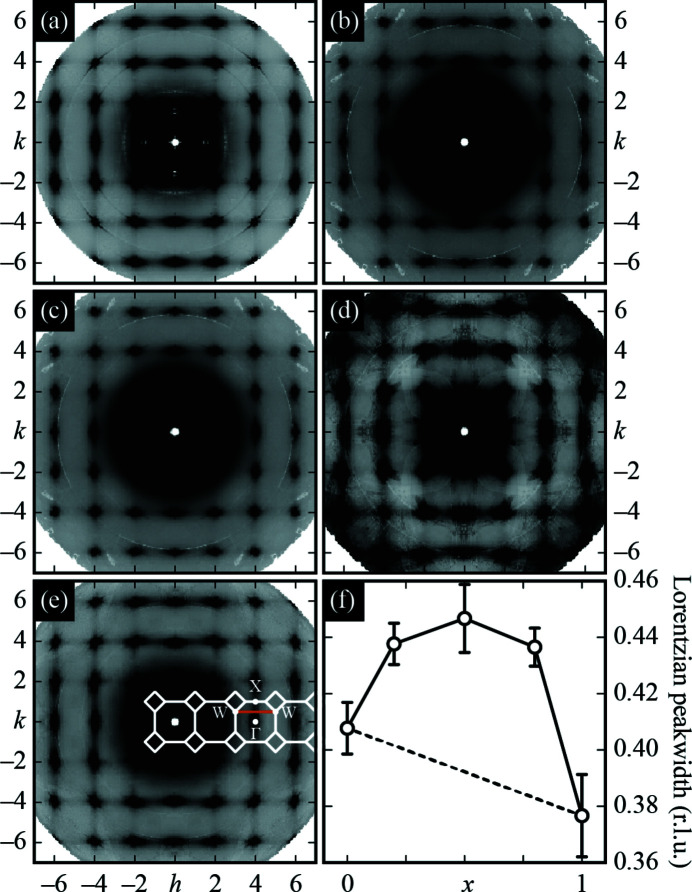
Experimental diffuse scattering and its compositional dependence. (*a*)–(*e*) Symmetry-averaged *hk*0 layers of the experimental single-crystal diffuse scattering patterns for KCl_1–*x*_Br_*x*_ with *x* = (*a*) 0.0, (*b*) 0.2, (*c*) 0.5, (*d*) 0.8 and (*e*) 1.0. Note the lines of diffuse scattering that connect neighbouring Bragg peaks along 〈100〉 directions. Selected Brillouin zone boundaries are shown schematically as white lines in panel (e), together with the position of key high symmetry positions, denoted using Bradley–Cracknell notation (Bradley & Cracknell, 1972 ▸). (*f*) Compositional dependence of the width of the Γ–X diffuse scattering feature determined using Lorentzian fits to the scattering profile measured along the W–Δ–W cut [orange line in panel (*e*)]. In the virtual crystal (mean-field) limit, the scattering function is expected to vary linearly with composition (dashed line). Further details of the fitting process are given as supporting information.

Our main experimental results are summarised in Figs. 1 ▸(*a*)–1 ▸(*e*), where we focus on the (*hk*0) sections of reciprocal space; a full map of the 3D diffuse scattering for the *x* = 0.5 crystal, which is representative of all data sets, is provided in the supporting information. For all five crystals we observe structured diffuse scattering that is of a single common form. This scattering includes strong broad maxima underneath the Bragg reflections, and weaker streaks of scattering that connect neighbouring Bragg reflections along the 〈100〉 directions. Some air and oil scattering is also clearly evident close to the centre of reciprocal space.

The form of this diffuse scattering has long been known for both KCl (Lonsdale & Smith, 1941 ▸; Hua *et al.*, 1989 ▸; Holm *et al.*, 2020 ▸) and KBr (Kashiwase & Kainuma, 1966 ▸), and has been attributed to thermal diffuse scattering from the low-energy transverse acoustic phonon branch along the Δ (Γ–X) direction in reciprocal space (*i.e.* **k** ∈ 〈ξ00〉) (Preston, 1939 ▸; Lonsdale & Smith, 1941 ▸; Kashiwase & Kainuma, 1966 ▸). Obviously there can be no short-range order or size-effect contributions to the diffuse scattering patterns of KCl or KBr. For the mixed-anion crystals, an additional size-effect contribution to the diffuse scattering was noted in an earlier X-ray diffuse scattering study (Luova *et al.*, 1970 ▸); the same study found no evidence, within the uncertainty of their measurements, for short-range compositional order. We will return to both points in due course.

While our observed diffuse scattering is qualitatively very similar for all our experiments, we are now in a position to quantify the subtle differences that do occur as a function of composition. To this end, we projected the diffuse scattering maps onto a single Brillouin zone (BZ) for each composition. In these projections, we observed a small but sensible variation in the width of the thermal diffuse scattering, which we can extract numerically using a Lorentzian fit to a cut in the scattering profile perpendicular to the Γ–X axis (we use the W–Δ–W 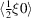 cut). These peakwidth values are unaffected by difference in intensity scale and/or (smoothly-varying) background contributions. Our data are shown in Fig. 1 ▸(*f*); further details of the fits involved are given as supporting information. The key observation is that the diffuse scattering broadens in **k**-space for intermediate compositions. We proceed to establish the origin of this broadening, which is unexpected in the virtual crystal (mean-field) limit.

### 2.3. Short-range order and associated lattice relaxations

*A priori*, we do not know whether it is the static diffuse scattering due to correlated substitutional disorder or variations in the lattice dynamics that dominate the additional diffuse scattering observed for the intermediate compositions. Our first step towards answering this question is to calculate the diffuse scattering patterns expected in the presence of different types of short-range compositional order, and subsequently how these patterns change when also taking into account the local lattice relaxation resulting from the different Cl^−^ and Br^−^ ionic radii.

Focusing on the KCl_0.5_Br_0.5_ composition, which shows the strongest broadening in experiment, we used a custom Metropolis Monte Carlo code to generate 10 × 10 × 5 supercells of the parent face-centred cubic unit cell (*i.e.* containing a total of 2000 halide ions). Assigning Ising states *e* = ±1 to Cl and Br atoms, the Monte Carlo energy was given by 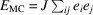 , where the sum is over nearest-neighbour anion sites and *J* could be tuned to favour either clustering (*J* < 0) or anticlustering (*J* > 0). By varying *J* and the effective Monte Carlo temperature, we could generate configurations with a range of short-range order strengths. We used the resulting Warren–Cowley short-range order parameter (Warren *et al.*, 1951 ▸) 

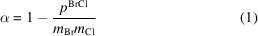

as a target in our simulations and to quantify the degree of local order. Here *p* ^BrCl^ is the probability of finding a Br–Cl pair on neighbouring anion sites, and *m*_Br_ and *m*_Cl_ are the relative concentrations of Br and Cl ( 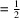 ). Our configurations spanned the range −0.2 ≤ α ≤ 0.8 (note the issue of geometric frustration on the f.c.c. lattice places strong constraints on the accessible values) and, in order to obtain better averaging, we generated 10 independent supercells for each α value.

**Figure 2:**
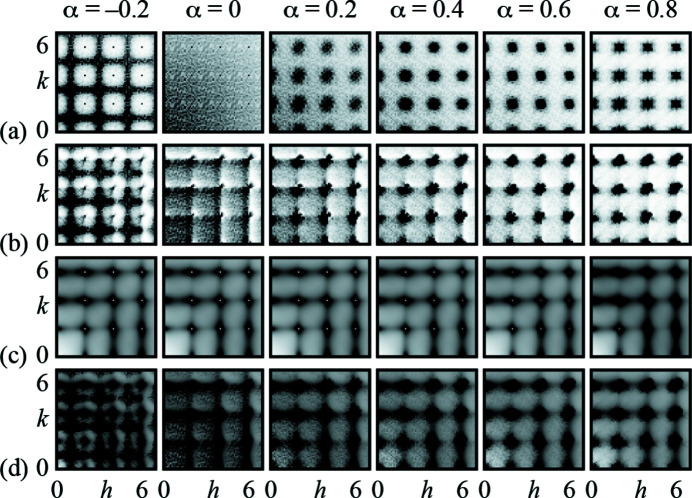
Sensitivity of the diffuse scattering function to short-range compositional order and its effects. (*a*) Calculated diffuse scattering patterns for KCl_0.5_Br_0.5_ considering only compositional order, as characterised by the Warren–Cowley parameter α (see text). Only one quadrant of the *hk*0 section is shown, such that the centre of reciprocal space lies at the bottom-left corner of each image. (*b*) Corresponding diffuse scattering patterns calculated after allowing relaxation of atomic positions according to the empirical potential reported in Catlow *et al.* (1977) ▸. (*c*) The corresponding thermal diffuse scattering patterns calculated using the SCLD approach described in the main text. (*d*) The total diffuse scattering function, obtained as the sum of static [panel (*b*)] and dynamic [panel (*c*)] contributions. We consider the match to experiment [Fig. 1 ▸(*c*)] to be closest for the α = 0.6, 0.8 values.

The corresponding X-ray diffuse scattering patterns, calculated using the DISCUS code (Neder & Proffen, 2008 ▸), are represented in Fig. 2 ▸(*a*). Anticlustering (α < 0) leads to structured diffuse scattering at the Brillouin zone boundary, which is not observed experimentally. By contrast, configurations with short-range clustering of Cl^−^ and Br^−^ ions give rise to diffuse scattering that is centred on the Bragg reflections. In the random (α = 0) case, the compositional diffuse scattering is incoherent and its smooth decay throughout reciprocal space reflects the difference in Cl and Br atomic form factors. Irrespective of the value of α, there is no structured diffuse scattering contribution to the Γ–X branch analysed above; hence the broadening effect identified in Fig. 1 ▸(*f*) cannot be understood in terms of simple compositional modulation.

The difference in ionic radii between Cl^−^ and Br^−^ results in a local lattice relaxation that also contributes to the diffuse scattering (Warren *et al.*, 1951 ▸; Welberry & Butler, 1994 ▸). To account for this additional contribution, we used the GULP lattice-dynamical code (Gale & Rohl, 2003 ▸; Gale, 2005 ▸) to relax our Monte Carlo configurations according to the empirical potentials developed for KCl and KBr in Catlow *et al.* (1977) ▸. The diffuse scattering patterns generated for these relaxed configurations are shown in Fig. 2 ▸(*b*); these now include all non-thermal contributions to the scattering function. The asymmetric scattering profiles we observe in these calculations are entirely consistent with size effects (Warren *et al.*, 1951 ▸), but are not obviously present in our experimental data. In particular, we can rule out the anticlustering and random scenarios, hence conclude α > 0, and must now determine the thermal diffuse scattering contribution.

### 2.4. Thermal diffuse scattering calculations

**Figure 3:**
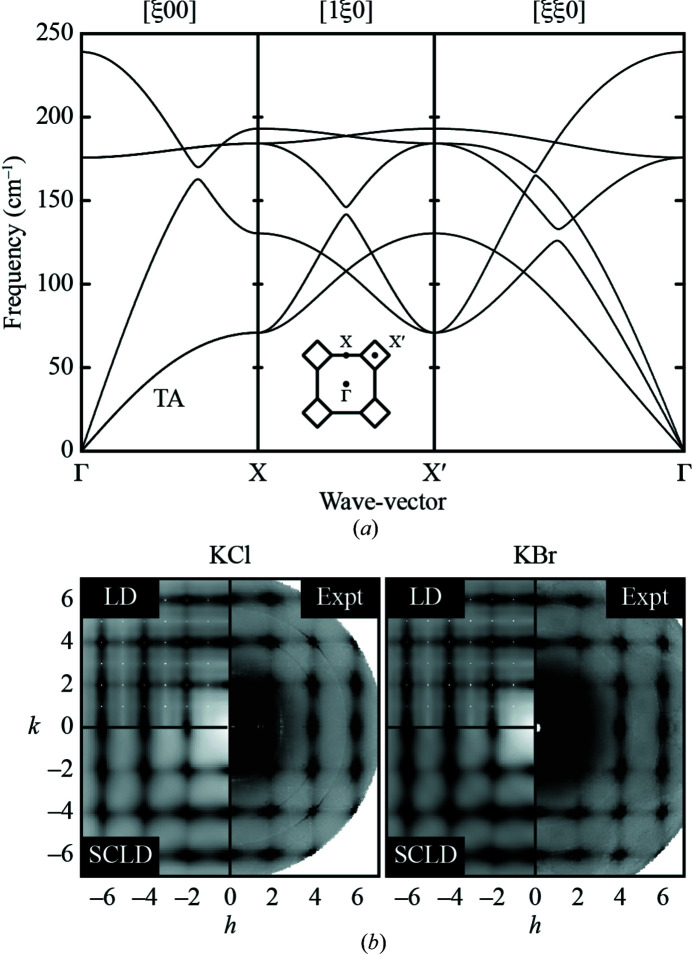
Lattice dynamics of KCl/KBr and its relationship to the thermal diffuse scattering contribution. (*a*) Harmonic phonon dispersion relation for KCl, calculated from the empirical potential of Catlow *et al.* (1977) ▸ using the GULP code (Gale & Rohl, 2003 ▸; Gale, 2005 ▸). Thermal diffuse scattering is dominated by the lowest-energy phonons, which correspond to the transverse acoustic (TA) branch along the Δ (Γ–X) line in reciprocal space. The dispersion relation for KBr is essentially identical, except that the phonon frequencies are lower as a consequence of the larger mass of Br relative to Cl. (*b*) Comparison of experimental diffuse scattering patterns for KCl and KBr with the thermal diffuse scattering calculated using conventional harmonic lattice dynamics (LD) and supercell lattice dynamics (SCLD). The close correspondence amongst the three functions indicates that (i) as expected, the diffuse scattering is purely dynamical in origin for these compositional end-members, and (ii) that our implementation of the SCLD thermal diffuse scattering calculations gives sensible results.

For the endmember compositions, it is straightforward to calculate the one-phonon thermal diffuse scattering using conventional lattice dynamical approaches. We first used the GULP code (Gale & Rohl, 2003 ▸; Gale, 2005 ▸) to determine the mode frequencies ω(**k**, ν) and eigenvectors **e**(**k**, ν) for all phonon branches ν across a suitable grid of wave vectors **k** within the first Brillouin zone. Here we are working within the harmonic approximation, and again using the empirical potentials of Catlow *et al.* (1977 ▸). The phonon dispersion curves we calculate for KCl are shown in Fig. 3 ▸(*a*); those of KBr are similar in form, but lower in energy as a consequence of the increased mass of Br *versus* Cl. Both results are entirely consistent with previous studies (Hua *et al.*, 1989 ▸; Copley *et al.*, 1969 ▸; Raunio & Almqvist, 1969 ▸). The thermal diffuse scattering is then given as (Dove, 1993 ▸; Bosak & Chernyshov, 2008 ▸; Xu & Chiang, 2005 ▸) 

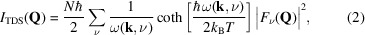

where *N* is the number of unit cells, ℏ is the reduced Planck’s constant, ν labels the phonon modes, *k*_B_ *T* is the Boltzmann temperature and **Q** = **G** + **k** relates the scattering vector and phonon wave vector via the nearest reciprocal lattice vector **G**. The one-phonon structure factor 

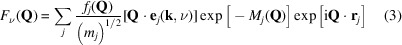

depends on the form factors *f*, masses *m*, Debye–Waller factors *M* and positions **r** of the atoms *j* in the unit cell (Trueblood *et al.*, 1996 ▸; Xu & Chiang, 2005 ▸). The corresponding diffuse scattering patterns calculated for KCl and KBr are compared against experiment in Fig. 3 ▸(*b*). The diffuse scattering agrees nicely with the data published by Holm *et al.* (2020) ▸. Note that the most intense streaks along 〈100〉 directions correspond to the low-energy transverse acoustic (TA) branch of the phonon dispersion.

Within the virtual crystal approximation, both the phonon dispersion and the calculated thermal diffuse scattering profile are given by a simple interpolation between the endmember results (Abeles, 1963 ▸). This is why the approximation is inconsistent with the nonlinear trend in diffuse scattering widths observed in experiment [Fig. 1 ▸(*f*)]. Consequently we must use supercell (band-unfolding) lattice dynamical calculations (Beltukov *et al.*, 2013 ▸; Larkin & McGaughey, 2013 ▸; Overy *et al.*, 2016, 2017 ▸) to determine the true effect of substitutional disorder on the thermal diffuse scattering.

To carry out these calculations, we developed a custom code that applied the supercell lattice dynamics (SCLD) formalism of Overy *et al.* (2017) ▸ to the calculation of thermal diffuse scattering from supercell configurations with arbitrary Cl/Br decorations. The basic premise is straightforward: the phonon frequencies and eigenvectors are determined using GULP for each of our Monte Carlo configurations but only at the Γ-point *of the supercell*. These values are then unfolded into the first Brillouin zone of the true (parent) cell onto a grid of **k**-points determined by the size of the supercell (large supercell = fine **k**-mesh), and the thermal diffuse scattering calculated using equations (2) and (3). For the disorder-free endmembers one expects identical results to conventional lattice dynamical calculations; indeed this is what we observe [Fig. 3 ▸(*b*)]. But it is also possible now to calculate the scattering expected for our various Monte Carlo configurations representing intermediate compositions.

Our results are shown in Fig. 2 ▸(*c*) for the specific composition KCl_0.5_Br_0.5_ and for each of the short-range order parameters α considered previously. While the form of the thermal diffuse scattering is essentially independent of α, we find that the width of the most intense features increases strictly monotonically with increasing α (here we use the same Lorentzian-fitting approach applied to our experimental data; see supporting information for further discussion). Hence the thermal diffuse scattering is directly sensitive to the extent and nature of short-range compositional order, despite the fact that the latter is a static, rather than dynamic, effect.

Summing together the various diffuse scattering contributions for each value of α gives the total diffuse scattering patterns shown in Fig. 2 ▸(*d*). Visual comparison of these patterns against the experimental result [Fig. 1 ▸(*c*)] suggests that the values 0.6 ≤ α ≤ 0.8 give the closest match. This implies a tendency for Cl^−^ and Br^−^ ions to cluster, at least within the samples we have studied here. We note that the study of Luova *et al.* (1970) ▸, which found no evidence for short-range compositional ordering within the sensitivity of their experimental measurements, used samples grown from the respective melts. The higher temperatures involved relative to our room-temperature crystallisations might favour anion disorder.

**Figure 4:**
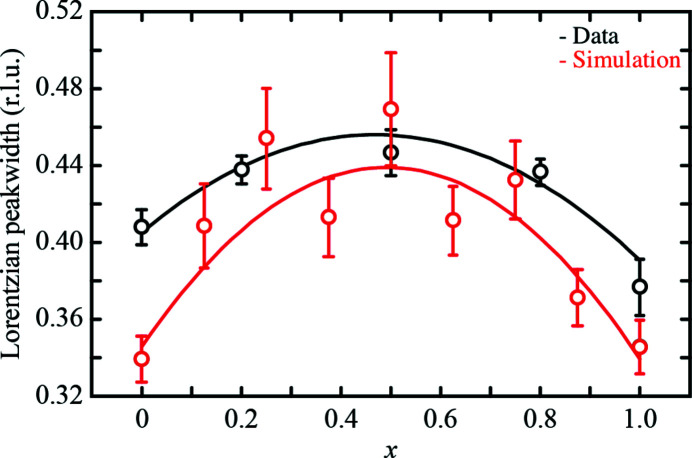
Compositional variation of the diffuse scattering width, determined using Lorentzian fits to the W–Δ–W scattering profiles calculated from our Monte Carlo configurations (short-range order parameter α = 0.7, red symbols) compared with the experimental data given in Fig. 1 ▸(*f*) (black symbols). The curves are error-weighted empirical fits of the form *f*(*x*) = (*ax* + *b*) + *c*[*x*(1 − *x*)], where the linear term represents a virtual-crystal (mean-field) contribution, and the binomial term captures the deviation that results from inhomogeneous mixing. The key result is that both experiment and calculation indicate broadened diffuse scattering features at intermediate compositions.

We repeated our thermal diffuse scattering calculations for a series of KCl_1–*x*_Br_*x*_ compositions, following precisely the workflow outlined above for the specific *x* = 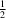 case and assuming a short-range order parameter value α = 0.7. Again we calculated the Lorentzian widths of the resulting diffuse scattering patterns using the same cuts described above. We show in Fig. 4 ▸ the evolution of diffuse scattering widths as a function of composition. We explain the considerable degree of scattering as a result of the relatively low number of supercells used for the static diffuse scattering calculations (see supporting information for further discussion). The experimental trend is nevertheless well accounted for by our calculations. Understandably, we have not taken into account the effects of instrumental resolution (independent of *x*) and crystal mosaicity, both of which mean we cannot expect quantitative match between experiment and calculation. Nevertheless we conclude from the overall trends we identify that the increase in diffuse scattering widths for intermediate KCl_1–*x*_Br_*x*_ compositions can be understood in terms of disorder-induced changes in the thermal diffuse scattering, and the interplay of this thermal component with the size-effect-induced local lattice relaxations.

### 2.5. Origin of phonon broadening

**Figure 5:**
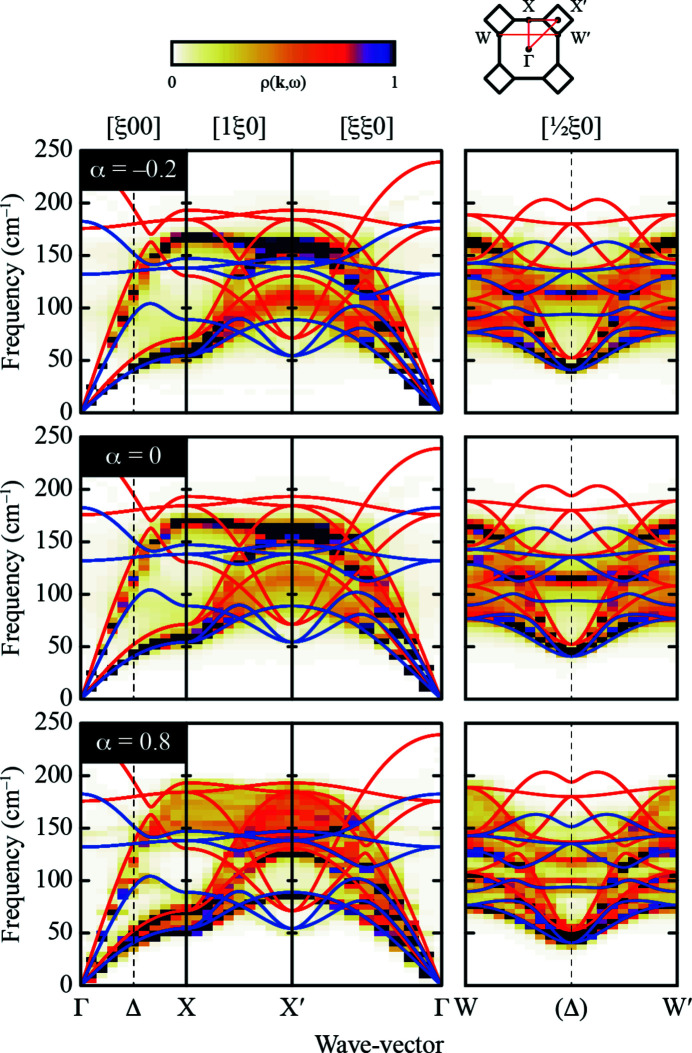
Effect of short-range compositional order on the phonon dispersion relations in KCl_0.5_Br_0.5_. Each panel shows the band-unfold SCLD projection ρ(**k**, ω) for key directions in reciprocal space and for different values of the short-range order parameter α. The W–Δ–W section shown on the right-hand side corresponds to the diffuse scattering cut used to calculate scattering peakwidths; its intersection with the Δ line is indicated by a dashed vertical line. The phonon dispersion relations for KCl and KBr are shown as red and blue lines, respectively. The virtual crystal approximation holds reasonably well for both α = −0.2 and 0: in most cases, the SCLD projection is well localised in both **k** and ω, and has its maxima at values intermediate to those for the KCl and KBr end-members. By contrast, the α = 0.8 projection shows very complex behaviour, with band-splitting and significant delocalisation in both **k** and ω. Note in particular the shallowness of the TA branch near 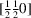 along the W–Δ–W line, which is responsible for the broader diffuse scattering measured experimentally.

To understand the origin of this disorder-driven phonon broadening, we interrogated the band-unfolded phonon dispersion curves obtained in our SCLD calculations for the intermediate *x* = 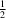 composition as a function of varying α. The dispersion functions along key directions in reciprocal space, including the W–Δ–W cuts used to determine diffuse scattering widths, are shown in Fig. 5 ▸. By virtue of the approximately inverse relationship between phonon frequency and thermal scattering intensity in equation (2), the thermal diffuse scattering is dominated by low-energy TA branch. Whereas for low and negative values of α this branch is well localised in both **k**- and frequency space, for larger positive values the branch begins to split and soften transverse to the Δ line. It is this softening that broadens the diffuse scattering, as picked up in our analysis.

In microscopic terms, the tendency for clustering associated with α > 0 short-range compositional order implies the formation of Cl^−^- and Br^−^-rich domains, as suggested also in Smakula *et al.* (1962) ▸. The boundaries between these domains act both to localise acoustic excitations in space (hence the broadening in **k**) and also to reduce phonon lifetimes (hence the broadening in ω). Both effects are evident in our calculated phonon dispersion curves.

## 3. Conclusion and outlook

Our key conclusion is that short-range compositional order in KCl_1–*x*_Br_*x*_ gives rise to local lattice relaxations that affect the lattice dynamics. Taken together, these various effects contribute to an increased broadening of structured diffuse scattering patterns observed in single-crystal X-ray diffuse scattering measurements. Intriguingly, it is the thermal diffuse scattering that is most strongly sensitive to Cl/Br short-range order; in this case because compositional order has a significant effect on the phonon dispersion.

An important corollary of this result is that control over anion ordering in KCl_1–*x*_Br_*x*_, *e.g.* by varying the synthesis temperature or quench rate, should enable control over phonon lifetimes. Since the acoustic phonons are almost entirely responsible for thermal transport in materials, this relationship allows in principle some targeted design of systems with low thermal conductivities (Zeuthen *et al.*, 2019 ▸; Roth *et al.*, 2021 ▸). This is an important consideration for thermoelectric materials, since the relevant figure of merit is maximised as thermal conductivity is reduced. We note many of the key thermoelectric materials are rocksalt structured (Zeuthen *et al.*, 2019 ▸), and that the spinodal texturing approach exploited in *e.g.* PbTe_0.7_Se_0.3_ (Wu *et al.*, 2014 ▸) can be associated with an extreme value (α → 1) of the same short-range ordering effects probed here. Recently, Zeuthen *et al.* (2022) ▸ studied PbS nanoinclusions in PbTe. While nanoinclusions can also be seen as an extreme value of α → 1, they suggest that the low-temperature thermal conductivity could be significantly influenced by strain fields, dislocations and point defects. After thermal cycling, which resulted in growth of the nanoinclusions and a strain reduction, the low-temperature thermal conductivity in their samples is increased. This result is consistent with our analysis, where we suggest that the broadening of the observed TDS with increasing α is caused by boundaries between Cl^−^ and Br^−^ rich domains which introduce strain at the domain boundaries and hence localise the acoustic excitations in space.

There is every reason to expect our results to generalise to the rocksalt family more generally. This is because the phonon broadening determined from our SCLD calculations effectively depends only on the degree of short-range compositional order, on the one hand, and the differences in interatomic potentials for different cation–anion pairs, on the other hand. Both aspects will vary from system to system, but there are no particular chemical requirements unique to the KCl_1–*x*_Br_*x*_ series. In general, we expect the most severe broadening effects in cases that favour anion clustering (α > 0), and with large differences in anion masses and/or effective cation–anion bond strengths.

As a final comment, we note that our results serve to reinforce the important point that in-house X-ray scattering measurements can provide surprisingly detailed insight into the lattice dynamics of solids (Bosak *et al.*, 2015 ▸). In this case, the SCLD approach allows one to draw an explicit link between compositional disorder and features of the phonon dispersion in a way that is not possible using conventional mean-field methods. We anticipate enormous benefit in applying similar methodologies to the study of a large variety of disordered functional materials for which lattice dynamics plays a crucial functional role (Simonov & Goodwin, 2020 ▸).

## 4. Related literature

The following references are cited in the supporting information: Sheldrick (2008 ▸), Coelho (2018 ▸), Paddison (2019 ▸).

## Supplementary Material

Additional information including Tables S1-S2 and Figs S1-S9. DOI: 10.1107/S2052520622003560/wu5006sup1.pdf
