# Supplementary material for: Interplay of thermal diffuse scattering and correlated compositional disorder in KCl1–x Br x
Source: Acta Crystallogr B Struct Sci Cryst Eng Mater. 2022 May 11;78(Pt 3):385–91. doi: 10.1107/S2052520622003560 (PMC9254590; doi:10.1107/S2052520622003560)
Supplement: Supplementary file 1 [file b-78-00385-sup1.pdf]

STRUCTURAL SCIENCE
CRYSTAL ENGINEERING
MATERIALS

Volume 78 (2022)

Supporting information for article:

Interplay of thermal diffuse scattering and correlated compositional disorder in $KCl_{1-x}Br_x$

Ella Mara Schmidt, Sofia Thomas, Johnathan M. Bulled, Arianna Minelli and Andrew L. Goodwin

Contents

1 Experimental details **S3**

Single crystal data . . . S3

Powder diffraction experiments . . . S4

2 Static disorder scattering calculations **S7**

3 Line width of the experimental diffuse scattering **S8**

4 TDS line width in $KBr_{0.5}Cl_{0.5}$ **S10**

5 TDS line width in KBr_xCl_{1-x} with $\alpha = 0.7$ **S11**

6 References **S12**

1 Experimental details

Single crystal data

The single crystals were picked from the glass containers and mounted onto the goniometer using MiTeGen loops. An example is shown in Figure S1. All crystals were thin plates with 100 -200 μm edge length and thicknesses between 20 and 50 μm.

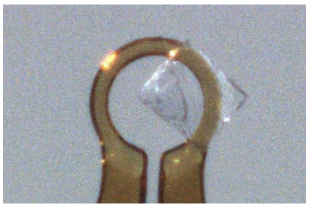

Figure S1: KBr thin plate crystal mounted onto the goniometer.

Diffraction experiments were conducted a Rigaku Synergy S diffractometer equipped with an Eiger 1M detector and using Cu radiation. For each sample two data sets were measured, one data set with short exposure times for Bragg diffraction analysis and one data set with longer exposure times for diffuse scattering data acquisition. For Bragg data analysis the data acquisition strategy as suggested by CrysAlisPro[S1] was used. For the diffuse scattering data collection we found the 'half sphere'-strategy most suitable. For all samples the sample-detector distance was 41 mm, 0.5° angular frames were taken with 60 to 120 s exposure, depending on Bragg intensities from the Bragg diffraction experiments.

Table S1: Lattice constants and refined compositions of the single crystal experiments of $KCl_{1-x}Br_x$.

Nominal x	a (Å)	Refined x
0	6.293(5)	-
0.2	6.439(3)	0.160
0.5	6.441(2)	0.429
0.8	6.520(3)	0.868
1	6.617(13)	-

Lattice constants were determined from Bragg diffraction measurements for all our specimens and we refined the composition of the mixed members using SHELXL.[S2] The lattice constants and the refined compositions are listed in Table S1 and show a reasonable agreement between the nominal and refined composition, especially taking into account the spread of reflections in the powder measurements reported below.

A three dimensional rendering of the observed diffuse scattering in $KCl_{0.5}Br_{0.5}$ is shown in Figure S2. We observe diffuse lines through the Bragg reflections and broad diffuse blobs underneath the Bragg reflections, as is clearly visible from the $hk0$-layer cuts in Figure 2 of the main text.

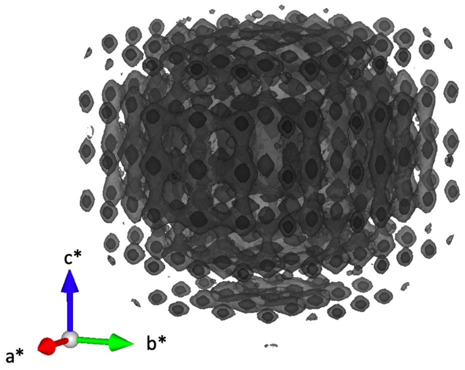

Figure S2: Three dimensional diffuse scattering in $KCl_{0.5}Br_{0.5}$.

In as K^+ and Cl^- ions have the same number of electrons, the (1 1 1) and the (3 3 3)-Bragg reflection are very weak compared to the (2,2,2)-Bragg peak in KCl. To assess the data quality of our diffuse scattering measurements line cuts along the ⟨1 1 1⟩- direction are shown in Figure S3. This underlines that our data quality is sufficient to do diffuse scattering analysis in reciprocal space.

Powder diffraction experiments

To further confirm the formation of the solid solution and to get an idea about the range of compositions within one synthesis, we perform powder diffraction measurements. The specimens were finely ground and diffraction experiments were performed on a Bruker D8 Advance Eco diffractometer using Cu radiation at ambient conditions. The diffraction data is shown in Figure S4 and was analysed with a Pawley refinement in Topas academic.[S3]

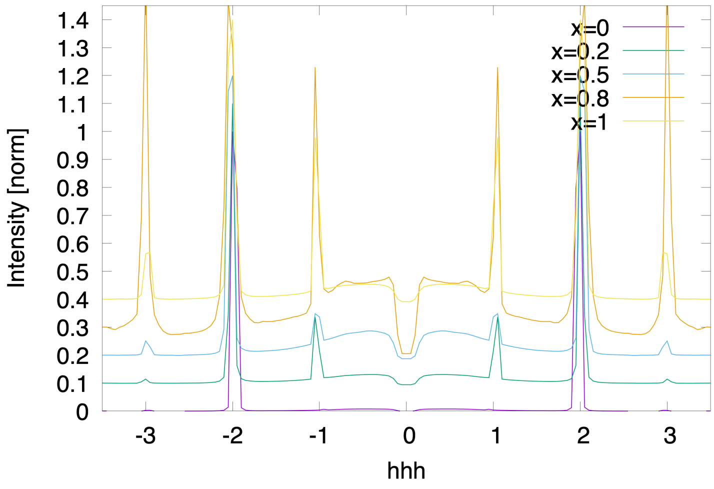

Figure S3: Line cuts of our symmetry averaged diffuse scattering data sets in the $\langle 1\ 1\ 1 \rangle$- direction. The curves are normalized to the intensity of the (2 2 2)-Bragg reflection and offset vertically for clarity.

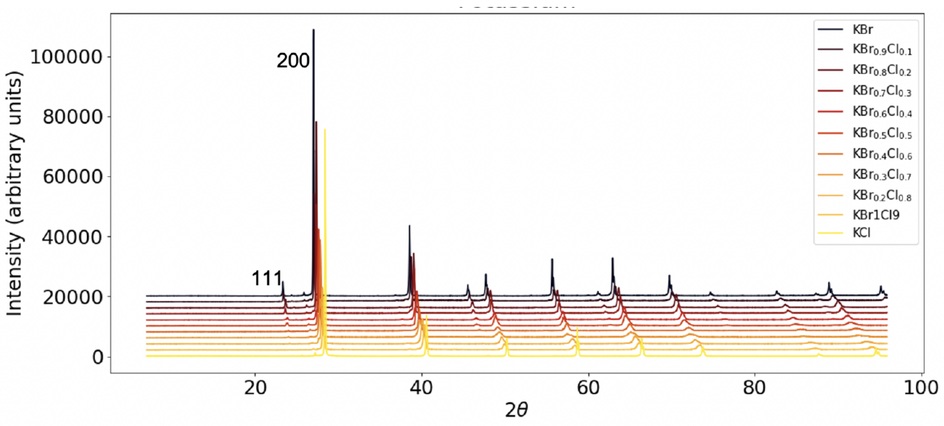

Figure S4: Powder diffraction measurements for the solid solution series $KCl_{1-x}Br_x$. The shift of the peaks indicates a change in lattice constants.

The lattice constants as a function of composition are depicted in Figure S5. The trend in lattice constants nicely follows Vegard's law. The peak width increases for the mixed members as compared to the end members, which we account for by two reasons: The synthesis will create a range of compositions, *i. e.* in the sample vial for $KBr_{0.5}Cl_{0.5}$ there will be a range of crystallites $KBr_{0.5-\delta}Cl_{0.5+\delta}$, which is consistent with the refined compositions in our single

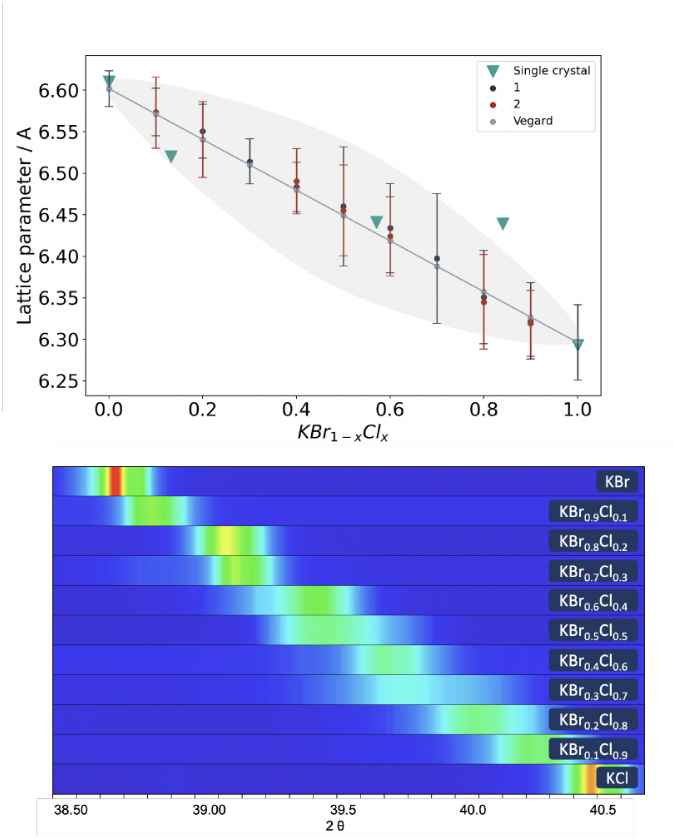

Figure S5: Top: Lattice constant as a function of composition for the solid solution series $KCl_{1-x}Br_x$ from single crystal experiments and powder diffraction measurements, compared to fit of Vegard's law. Error bars represent the lattice constant variation calculated from the FWHM of the peak width of the (2,0,0)-peak. Bottom: Normalised waterfall film plots of the (2,2,0)-peak showing the variations in peak widths and lattice constant.

crystals (see Table S1). Another effect that we need to take into account is possible strain in the mixed members, which is known for this system in literature.[S4] Overall these measurements confirm the formation of the solid solution series and show that the compositions we determined from the single crystal refinements are reasonable.

2 Static disorder scattering calculations

The calculations of the static scattering were performed using DISCUS.[S5] The generation and relaxation of the supercells employs periodic boundary conditions and these are also used in the calculation of the diffraction pattern. To avoid the effects of Fourier ripples, the same grid in reciprocal space as for the supercell lattice dynamical calculations is chosen ($\Delta h = \Delta k = 0.1$). In the simulation the Bragg scattering is not subtracted, but as Bragg reflections only span one pixel in our simulation and the cuts we take to analyse the width of the diffuse scattering are far from the Bragg reflections, this does not effect our subsequent analysis.

While it is possible to compute smoother and finer sampled diffuse scattering pattern, e.g. using the lots algorithm[S5] or sampling over many more disordered supercells,[S6] we intentionally chose to not use these tools, to employ the same statistical averaging for the thermal and static diffuse scattering. In our subsequent analysis we observe that the calculated static diffuse scattering seems to be subject to more noise than the TDS calculations from SCLD calculations. For a complete analysis of the total diffuse scattering with sufficient statistics, we would suggest further supercell simulations or the simulation of larger supercells. Due to the computationally expensive structure relaxation and SCLD calculations these were not performed in this study.

3 Line width of the experimental diffuse scattering

While our observed diffuse scattering is qualitatively very similar for all our experiments, we seek to quantify the differences in the diffuse scattering as a function of composition. To quantify these differences we took cuts of the data along the six symmetry equivalent $\langle \zeta, \frac{1}{2} + n, 2m \rangle$ $(n, m \in \mathbb{Z})$ directions. The observed intensities were then averaged over all n and m, projected into one Brillouin zone and normalized to have minimum value 0 and maximum value 1. To avoid artificial broadening due to potential lattice misfits in the reconstructions these cuts were taken before symmetry averaging the data. Figure S6 shows the cuts for one specific member, $KBr_{0.2}Cl_{0.8}$.

Figure S6: Cuts through the diffuse scattering of $KBr_{0.2}Cl_{0.8}$ along $\langle \zeta\ \frac{1}{2}\ 0 \rangle$. The intensity is projected into one Brillouin zone $(0 \leq \zeta < 2)$ and normalized to the intensity range 0 to 1. The intensity is repeated for $2 \leq \zeta \leq 4$ to show the peak shape that is fitted with the Lorenzian in Equation 1 for the range $1 \leq \zeta \leq 3$. Fits are shown as solid lines. For more details see text.

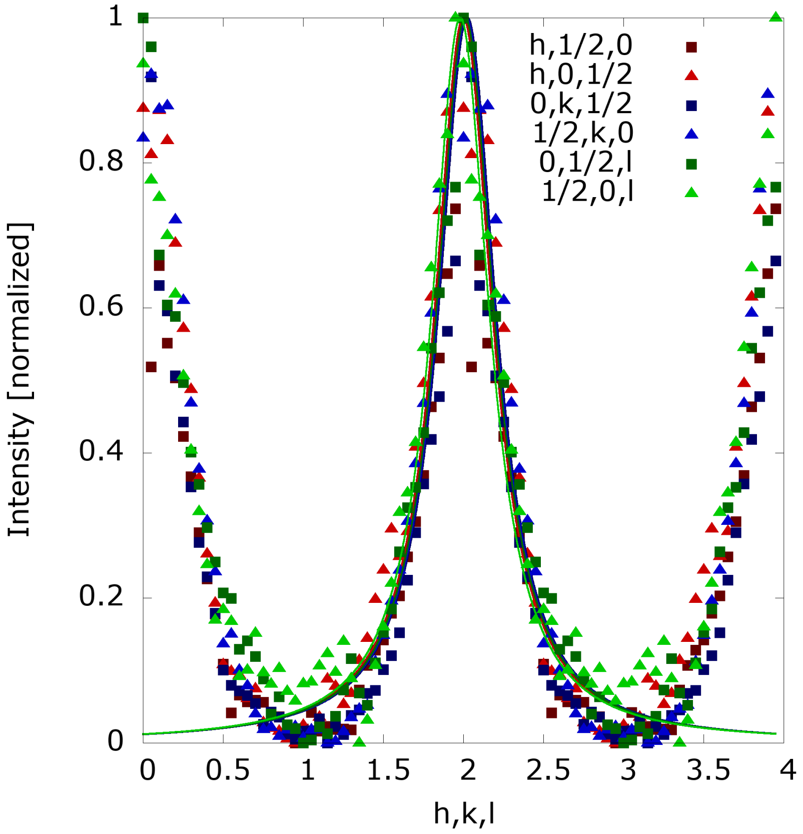

The broad diffuse background visible in Figure 1 of the main manuscript does not affect our analysis. The projection of the broad background intensity into one Brillouin zone essentially yields a flat background, which is subtracted in our subsequent normalization.

To quantify the width of the observed diffuse streaks the resulting cuts in Figure S6 were fitted using a Lorentzian with maximum intensity 1:

$$I(h) = \frac{\gamma^2}{4(\zeta - \zeta_0)^2 + \gamma^2}, \tag{1}$$

where ζ_0 describes the centre of the distribution and γ is a parameter that describes the width of the distribution. The fit was restricted to the range $1 \leq \zeta \leq 3$, which covers the range of one Brillouin zone and includes the complete peak. For all cuts of a given composition x, we refine the same peak width γ but allow ζ_0 to vary, to compensate for possible uncertainties and misfits in the reconstruction of the three-dimensional data. The resulting fits are shown in S6 as the solid lines and describe the experimentally observed line shapes very well.

The plot shown in Fig.1(f) of the main paper shows the fitted diffuse scattering line width γ in reciprocal lattice units as a function of composition. The error bars represent the error from the least-squares refinement procedure. We observe a strong increase in the observed width of the diffuse scattering for the mixed members as compared to the end members.

4 TDS line width in $KBr_{0.5}Cl_{0.5}$

Figure S7 shows the cuts $\langle \zeta, \frac{1}{2} + n, 0 \rangle$ ($n \in \mathbb{Z}$) for the TDS simulations for different degrees of local order α in $KBr_{0.5}Cl_{0.5}$. Both fit and data show a clear broadening of the observed diffuse line width as a function of composition.

Figure S7: Cuts $\langle \zeta, \frac{1}{2} + n, 0 \rangle$ ($n \in \mathbb{Z}$) of the TDS (row 3 in Figure 3 of the main text) fitted with a Lorentzian peak.

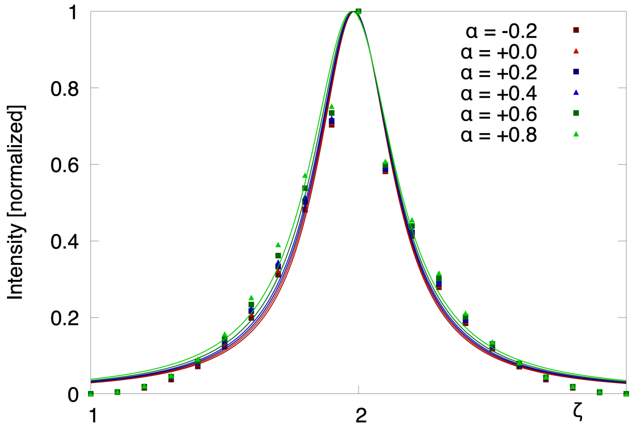

The compositional trend of Lorentzian peakwidth is shown explicitly in Fig. S8.

Figure S8: Width γ of the first order TDS for $KCl_{0.5}Br_{0.5}$ as a function of short range order parameter α.

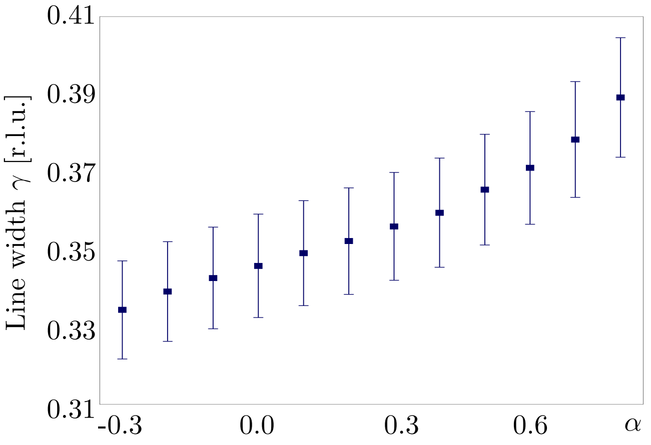

5 TDS line width in KBr_xCl_{1-x} with $\alpha = 0.7$

The TDS line width of the first order diffuse scattering as a function of composition is shown in Figure S9. We observe a significant broadening of the TDS for the mixed members. The broadening due to TDS accounts for about half the total broadening of the total diffuse scattering, see fit parameters listed in Table S2.

Figure S9: Lorentzian peak width γ of the first order TDS for KCl_xBr_{1-x} as a function of composition x compared to the experiment and total diffuse scattering width.

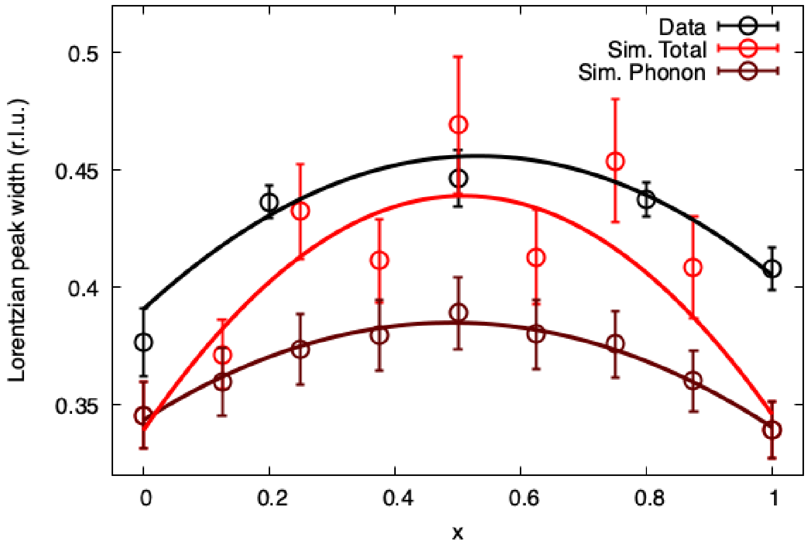

Table S2: Fit parameters of $f(x) = (ax+b)+c[x(1-x)]$ for the experiment, total diffuse scattering and pure thermal diffuse scattering.

Parameter	Experiment	Total diffuse scattering	pure TDS
a	0.0145 ± 0.0129	0.0068 ± 0.0182	-0.0030 ± 0.0026
b	0.3907 ± 0.012	0.3391 ± 0.0141	0.3434 ± 0.0022
c	0.2312 ± 0.0555	0.3859 ± 0.0717	0.1721 ± 0.0094

Raw data, data reconstructions, supercell simulation boxes and phonon eigenvalues and eigenvectors can be provided upon request.

6 References

(S1) Agilent, (2014). CrysAlis PRO. Agilent Technologies Ltd, Yarnton,Oxfordshire, England.

(S2) Sheldrick, G. M. (2008). *Acta Cryst. A*, **64**, 112–122.

(S3) Coelho, A. A. (2018). *J. Appl. Cryst.*, **51**, 210-218.

(S4) A. Smakula, N. Maynard & A. Repucci. (1962) *J. Appl. Phys.*, **33**, 453–455.

(S5) Neder, R. B. & Proffen, T. (2008). *Diffuse Scattering and Defect Structure Simulations*, vol. 11. Oxford University Press.

(S6) Paddison, J. A. M. (2019). *Acta Cryst.* **A75**(1), 14–24.
